# A Superoxide Dismutase Capable of Functioning with Iron or Manganese Promotes the Resistance of *Staphylococcus aureus* to Calprotectin and Nutritional Immunity

**DOI:** 10.1371/journal.ppat.1006125

**Published:** 2017-01-19

**Authors:** Yuritzi M. Garcia, Anna Barwinska-Sendra, Emma Tarrant, Eric P. Skaar, Kevin J. Waldron, Thomas E. Kehl-Fie

**Affiliations:** 1 Department of Microbiology, University of Illinois Urbana-Champaign, Urbana, IL, United States of America; 2 Institute for Cell and Molecular Biosciences, Newcastle University, Newcastle upon Tyne, United Kingdom; 3 Department of Pathology Microbiology and Immunology, Vanderbilt University Medical Center Nashville TN, United States of America; University of Tubingen, GERMANY

## Abstract

*Staphylococcus aureus* is a devastating mammalian pathogen for which the development of new therapeutic approaches is urgently needed due to the prevalence of antibiotic resistance. During infection pathogens must overcome the dual threats of host-imposed manganese starvation, termed nutritional immunity, and the oxidative burst of immune cells. These defenses function synergistically, as host-imposed manganese starvation reduces activity of the manganese-dependent enzyme superoxide dismutase (SOD). *S*. *aureus* expresses two SODs, denoted SodA and SodM. While all staphylococci possess SodA, SodM is unique to *S*. *aureus*, but the advantage that *S*. *aureus* gains by expressing two apparently manganese-dependent SODs is unknown. Surprisingly, loss of both SODs renders *S*. *aureus* more sensitive to host-imposed manganese starvation, suggesting a role for these proteins in overcoming nutritional immunity. In this study, we have elucidated the respective contributions of SodA and SodM to resisting oxidative stress and nutritional immunity. These analyses revealed that SodA is important for resisting oxidative stress and for disease development when manganese is abundant, while SodM is important under manganese-deplete conditions. *In vitro* analysis demonstrated that SodA is strictly manganese-dependent whereas SodM is in fact cambialistic, possessing equal enzymatic activity when loaded with manganese or iron. Cumulatively, these studies provide a mechanistic rationale for the acquisition of a second superoxide dismutase by *S*. *aureus* and demonstrate an important contribution of cambialistic SODs to bacterial pathogenesis. Furthermore, they also suggest a new mechanism for resisting manganese starvation, namely populating manganese-utilizing enzymes with iron.

## Author Summary

During infection, pathogens must overcome the restriction of essential nutrients such as manganese by the host, while simultaneously coping with other host defenses such as the oxidative burst. Using the host protein that limits manganese availability during infection and mice lacking this effector, we determined that acquisition of a second superoxide dismutase that is capable of using either manganese or iron enhances the ability of *Staphylococcus aureus* to cause infection. When manganese-starved by the host, this cambialistic enzyme enables *S*. *aureus* to maintain superoxide dismutase activity and survive when exposed to oxidative stress. These results reveal the important contribution of cambialistic superoxide dismutases to bacterial pathogenesis and represent a new mechanism for resisting manganese starvation during infection.

## Introduction

The spread of antibiotic resistance amongst bacteria has led both the Centers for Disease Control and Prevention and the World Health Organization to state that infections represent a serious threat to human health [1, 2]. This threat is exemplified by *Staphylococcus aureus*, a Gram-positive bacterium that asymptomatically colonizes one third of the population and is a leading cause of antibiotic-resistant infections [3–5]. A promising area of investigation is elucidating how pathogens overcome host defenses such as the active withholding of essential nutrients and the oxidative burst of immune cells.

During infection, pathogens must obtain all of their nutrients from the host, including the essential metal ions that are needed for the approximately one-third of all bacterial proteins that require a metal cofactor [6–8]. This requirement is exploited by the host, which restricts the availability of these essential nutrients, a defense termed nutritional immunity [9–13]. The canonical example of nutritional immunity is the iron (Fe)-withholding response [10, 11]. In addition to Fe, the host also restricts the availability of manganese (Mn) and zinc (Zn) [9, 12–14]. The prototypical example of Mn and Zn restriction is the staphylococcal abscess, which is rendered virtually free of these metals during infection [9, 14]. A critical component of the Mn- and Zn-withholding response is the host protein calprotectin (CP) [9, 12, 14]. This innate immune effector is highly expressed in neutrophils in which it comprises 40–60% of the cytoplasmic protein, and at sites of infection it can be found in excess of 1 mg/ml [15, 16]. Loss of CP results in host defects in metal sequestration and increased sensitivity to a number of bacterial and fungal pathogens, including *S*. *aureus* [9, 14, 17–19]. In culture, CP inhibits the growth of a similarly wide range of pathogens [16–20]. The antimicrobial activity of CP is dependent on binding of metal ions to its two transition metal-binding sites [14, 20, 21]. The first site or ‘Mn/Zn site’ is comprised of six histidines and is capable of binding either Mn or Zn with nanomolar and picomolar affinities (*K*_d_), respectively [14, 20–22]. The second site or ‘Zn site’ is comprised of three histidines and an aspartic acid and binds Zn with picomolar or sub-picomolar affinity [14, 20, 22].

In order to cause disease, invading pathogens must not only overcome nutrient starvation but also simultaneously cope with other host defenses, such as the oxidative burst of neutrophils and other immune cells [23]. Bacteria defend themselves from the oxidative burst by numerous mechanisms, including enzymes such as superoxide dismutases (SODs) that detoxify the damaging reactive oxygen species with which they are bombarded [24–27]. The activating metal cofactor divides the SOD enzymes into several families, with the most common amongst bacteria belonging to a single protein superfamily, which utilizes either Mn or Fe as cofactor. It has proven exceptionally difficult to predict which metal is utilized by a given Mn/Fe-dependent SOD [24, 28], in part due to the fact that Fe- and Mn-SODs coordinate their metal cofactor using the same protein ligands within an identical protein fold [28, 29]. This structural similarity also enables both Fe- and Mn-SODs to bind the other, non-cognate metal, but this usually results in an inactive enzyme; most members of this protein family are strictly dependent on their cognate metal for catalysis [28–31]. Notably, a subset of the Mn/Fe-dependent SOD family is active when loaded with either Fe or Mn [32–40]. While these ‘cambialistic’ SODs are present in a diverse group of microbes, analysis of their activity has largely been limited to *in vitro* studies, limiting our understanding of how cambialism benefits microbes and the contribution of these enzymes to colonization of the host.

*S*. *aureus* possesses two SODs, SodA and SodM, both of which are cytoplasmic and are reported to be Mn-dependent [41–43]. While all staphylococci possess SodA, SodM is unique to *S*. *aureus* [44]. Highlighting their importance to virulence, loss of either SodA or SodM in a skin model of infection or loss of both SODs in a systemic mouse model, reduces the ability of *S*. *aureus* to cause disease [14, 42]. However, a molecular explanation for the advantage that *S*. *aureus* gains by expressing two apparently Mn-dependent SODs is unknown. Host-imposed Mn starvation mediated by CP reduces total staphylococcal SOD activity, both in culture and during infection, which renders *S*. *aureus* more sensitive to oxidative stress and neutrophil-mediated killing [13, 14, 20]. Yet paradoxically, the simultaneous loss of both SodA and SodM renders *S*. *aureus* more sensitive to CP [14], indicating that SodA and/or SodM somehow enhance the ability of *S*. *aureus* to resist metal starvation.

Given the importance of the two staphylococcal SODs to infection, we have elucidated their respective contributions to resisting oxidative stress and nutritional immunity. This analysis revealed that SodA is important for resisting oxidative stress and infection when Mn is abundant, whereas SodM is important under Mn-deplete conditions. Our data demonstrate that SodM is in fact cambialistic, possessing equal enzymatic activity when loaded with Mn or Fe. We propose that the ability of SodM to utilize Fe enables *S*. *aureus* to retain SOD activity when starved of Mn by the host, thereby enhancing the ability of the bacterium to overcome nutritional immunity, resist oxidative stress, and ultimately cause infection.

## Results

### Mn availability differentially regulates the expression of the *S*. *aureus* SODs

**Fig 1:**
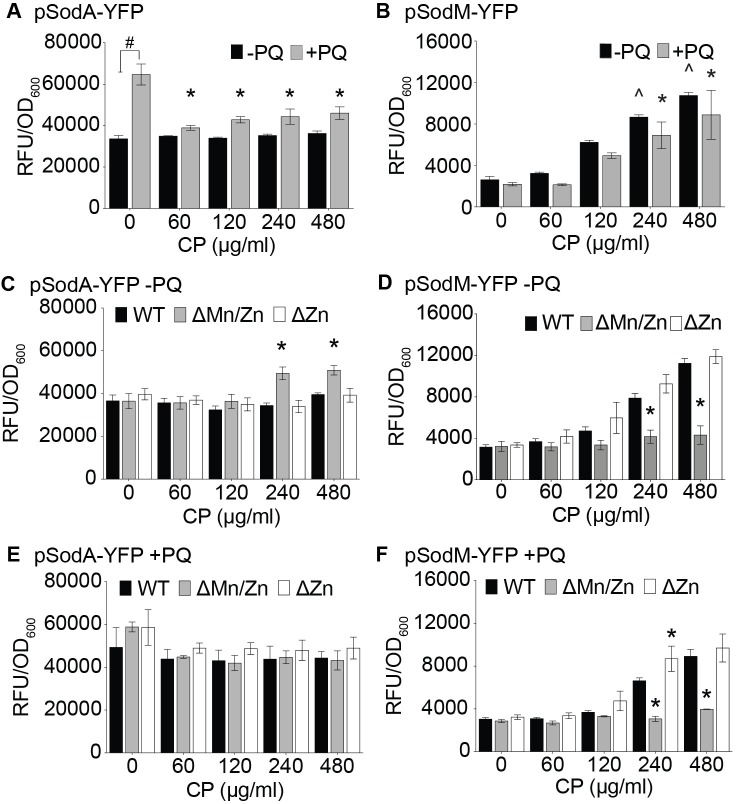
Expression of *sodA* and *sodM* varies by Mn availability. *S*. *aureus* carrying (A, C, & E) pSodA and (B, D, & F) pSodM YFP reporter constructs were grown in the presence of either wild type CP (A-F), the ΔMn/Zn site mutant (C-F), or the ΔZn site mutant (C-F), and in the (A, B, E, & F) presence or (A-D) absence of 0.1 mM PQ. Expression data are normalized to growth. Error bars indicate SEM (n = 3 or more). (A & B) # = p <0.05 via two-way ANOVA with Tukey’s post-test for the indicated comparison. * = p <0.05 via two-way ANOVA with Tukey’s post-test relative to bacteria grown in the presence of paraquat without CP. ^ = p <0.05 via two-way ANOVA with Tukey’s post-test relative to bacteria grown in the absence of both paraquat and CP (C-F) * = p <0.05 via two-way ANOVA with Dunnett’s post-test when compared to wild type CP.

Several prior studies have examined the impact of Mn availability on the expression of SodA and SodM; however, different conclusions were reached [41, 42, 45]. In light of this ambiguity, we initially assessed the impact that CP and oxidative stress have on *sodA* and *sodM* expression. When normalized to optical density in order to account for differences in growth, high levels of *sodA* transcription were observed regardless of whether CP was present (Fig 1A), whereas the presence of CP enhanced *sodM* expression independent of the presence of oxidative stress (Fig 1B). These results indicate that *sodM*, but not *sodA*, is induced in response to Mn or Zn limitation. To clarify which metal gave rise to this effect, we used mutant CP variants that lack either the Mn/Zn site (ΔMn/Zn site mutant, which does not bind Mn) or the Zn site (ΔZn site mutant, which binds both Mn and Zn) [20]. As expected, neither mutant induced the expression of *sodA* (Fig 1C). The increased expression of *sodM* observed with wild type (WT) CP is lost in the presence of the ΔMn/Zn site mutant, but not the ΔZn site mutant, indicating that *sodM* is induced in response to Mn limitation (Fig 1D).

We also evaluated the impact of oxidative stress on the expression of the SODs using the superoxide-generating compound paraquat (PQ). Similar to previous studies [42], PQ induced the expression of *sodA* in metal-replete media (Fig 1A). However, PQ did not alter the induction of expression of *sodM* observed with CP (Fig 1B), nor did it change the *sodA* and *sodM* expression pattern observed with the ΔMn/Zn or ΔZn site mutants (Fig 1E & 1F). Cumulatively, these observations suggest that the expression of SodM increases when *S*. *aureus* is Mn-limited regardless of level of oxidative stress experienced by *S*. *aureus*.

### The activity of the staphylococcal SODs does not correlate with gene expression

**Fig 2:**
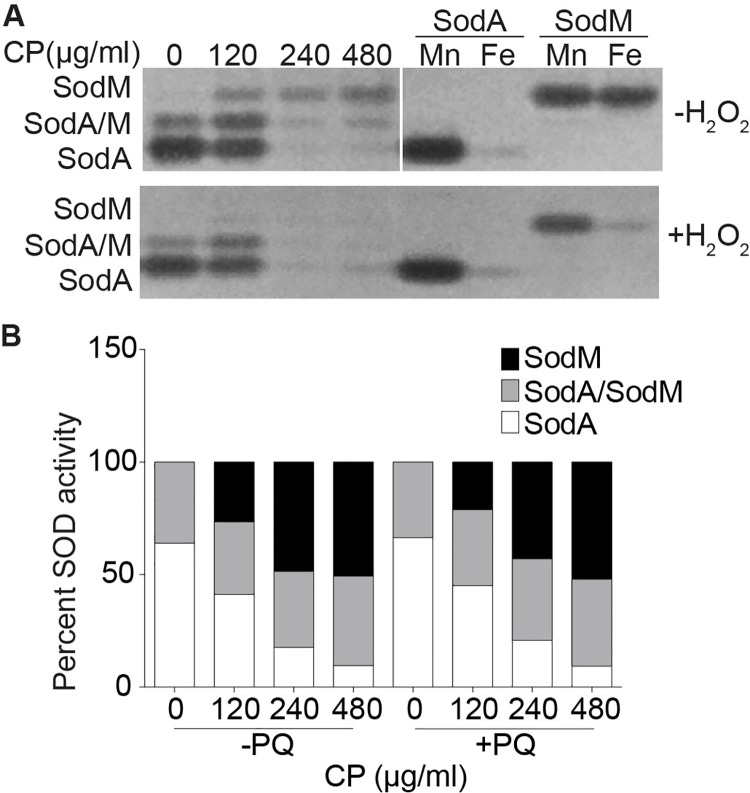
SodM is the predominant source of SOD activity in Mn-deplete environments. Wild type *S*. *aureus* was grown in various concentrations of CP in the (A & B) absence and (B) presence of 0.1 mM PQ, and both (A) the individual activities of the SODs and (B) the fractional contribution of SodA and SodM to total SOD activity in cell lysates (5.17 μg of total protein) was determined. The lower gel was treated with hydrogen peroxide prior to assessing SOD activity to inactivate Fe-containing SODs. Purified recombinant SodA and SodM (0.3 μg), loaded with either Mn or Fe, were included as controls. The experiment was repeated 3 times and representative gels are shown.

The propensity of Mn/Fe-SODs to acquire the wrong metal can result in discordance between expression levels and enzymatic activity [46]. In order to determine if SodA and SodM activity correlated with expression, total and individual SOD activity were assessed in the presence and absence of CP and PQ. Consistent with prior results, CP significantly reduced total staphylococcal SOD activity [14] (S1A Fig). In the absence of CP, the predominant activity comes from SodA (Fig 2A & 2B). The presence of the SodA/SodM heterodimer [41, 43] indicates that SodM is present in Mn-replete media, although the SodM homodimer’s activity is barely detectable in the gel. In the presence of CP, the relative contribution of SodA to total SOD activity decreased while that of SodM increased (Fig 2A & 2B). Notably, not only did the fractional contribution of SodM change, the absolute level of SodM activity also increased (Figs 2A & S1C). Consistent with prior studies, the addition of PQ increased total staphylococcal SOD activity [14] (S1A Fig). However, the addition of PQ did not change the impact that Mn availability had on the relative contributions of SodA and SodM to total staphylococcal SOD activity (Figs 2B, S1B & S1C). Together, these results indicate that in Mn-replete environments SodA is the primary source of SOD activity but SodM becomes the predominant SOD when *S*. *aureus* experiences Mn starvation.

### SodA and SodM differentially contribute to resisting oxidative stress based on Mn availability

**Fig 3:**
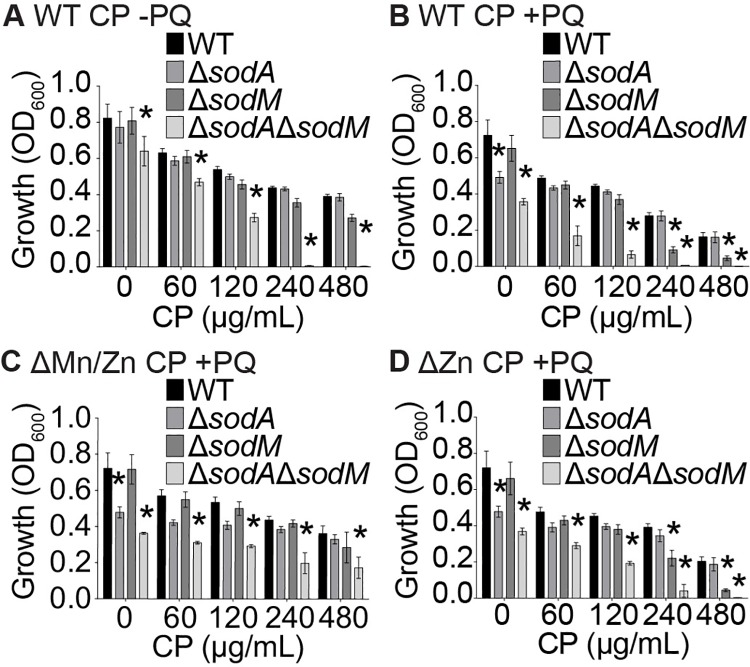
SodM is required to resist CP-imposed Mn starvation. Wild type, *ΔsodA*, *ΔsodM* and Δ*sodA*Δ*sodM S*. *aureus* were grown in the presence of increasing concentrations of (A & B) WT CP, (C) the ΔMn/Zn site mutant, or (D) the ΔZn site mutant in the (A) absence and (C-D) presence of 0.1 mM PQ. Growth was assessed by measuring optical density (OD_600_). * = p<0.05 relative to wild type at the same concentration of CP via two-way ANOVA with Dunnett’s post-test. Error bars indicate SEM (n = 3 or more).

To test the respective contribution of each SOD to resisting Mn starvation, the ability of Δ*sodA* and Δ*sodM* single mutants, as well as a Δ*sodA*Δ*sodM* double mutant, to grow in the presence of CP was assessed. Similar to previous results [14, 42], Δ*sodA*Δ*sodM* was profoundly more sensitive to CP and PQ than wild type (Fig 3A & 3B), while ectopic expression of either SodA or SodM reversed this sensitivity (S2 Fig). In the absence of PQ, Δ*sodA* grew as well as wild type *S*. *aureus* in both the presence and absence of CP (Fig 3A), whereas the Δ*sodM* mutant, although it did not reach significance, displayed consistent reduced growth relative to wild type at high levels of CP (Fig 3A). Given the role of SodA and SodM in detoxifying superoxide, we also evaluated the impact of Mn availability on the ability of Δ*sodA* and Δ*sodM* to resist oxidative stress. Consistent with the activity analysis and its reported role as the primary SOD expressed by *S*. *aureus* [41, 42], loss of SodA resulted in increased sensitivity to PQ in the absence of CP (Fig 3B). However, at high concentrations of CP Δ*sodA* was no more sensitive to PQ than wild type *S*. *aureus* (Fig 3B). When compared to Δ*sodA*, the impact that CP had on the sensitivity of Δ*sodM* to oxidative stress was reversed; the Δ*sodM* mutant was no more sensitive to PQ than wild type bacteria in the absence and presence of low concentrations of CP, but the mutant was significantly more sensitive at high concentrations (Fig 3B). Notably, in the presence of intermediate CP concentrations in which both SodA and SodM are active, neither Δ*sodA* nor Δ*sodM* is more sensitive than WT *S*. *aureus* to oxidative stress. Utilization of the CP metal-binding site mutants revealed that both in the presence and absence of PQ, the increased sensitivity of Δ*sodM* is due to Mn sequestration (Figs 3C, 3D, S2C & S2D). Cumulatively, these results indicate that in Mn-replete environments SodA is primarily responsible for protecting *S*. *aureus* from oxidative stress, whereas SodM is critical for protecting *S*. *aureus* from oxidative stress in Mn-deplete environments. They also suggest that SodM promotes resistance to nutritional immunity by facilitating the retention of SOD activity and resistance to oxidative stress.

### SodM is cambialistic

Paradoxically, our results indicate that the reportedly Mn-dependent enzyme SodM promotes resistance to host-imposed Mn starvation. In light of these observations, we analyzed the metal specificities of recombinant SodA and SodM. To facilitate these studies, the SODs were expressed in and purified from *E*. *coli* grown in iron-replete media. Following expression in *E*. *coli* and consistent with negligible Mn accumulation by *E*. *coli* in the absence of oxidative stress [46], inductively coupled plasma mass spectrometry (ICP-MS) analysis revealed that both of the purified recombinant staphylococcal SODs were loaded with Fe when recovered from the heterologous host (S3 Fig). Substantial activity was observed with purified Fe-SodM (210 +/- 21 U/mg protein), but negligible activity was detected from Fe-SodA (4 +/- 1 U/mg). Each of the recombinant proteins were denatured in the presence of metal chelators and then refolded in the presence of Mn *in vitro*, with successful elimination of Fe and loading with Mn confirmed by ICP-MS (S3 Fig). Enzymatic analysis revealed that the Mn-SodA form has substantial activity (1594 +/- 81 U/mg) in contrast to the Fe form. Surprisingly, Mn-SodM was also active (215 +/- 21 U/mg) and to a degree similar to that of the Fe-SodM, although both forms display activity substantially lower than that of Mn-SodA. The comparable activity of the Mn- and Fe-loaded forms of SodM indicate that it is not Mn-dependent, as previously suggested, but cambialistic [43].

### Cambialism enables SodM to retain activity when *S*. *aureus* is Mn-starved

**Fig 4:**
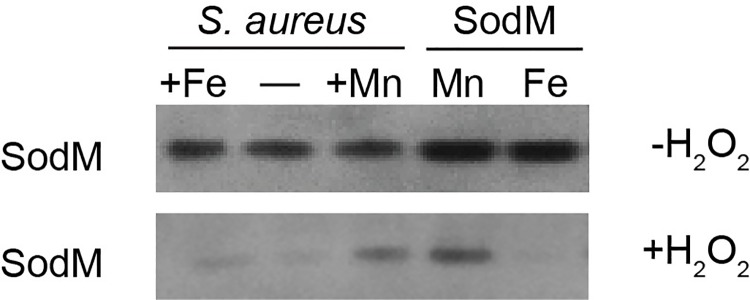
SodM can be metallated with either Mn or Fe in *S*. *aureus*. The *S*. *aureus* Δ*sodA*Δ*sodM* mutant expressing SodM from a plasmid was grown in NRPMI supplemented with either 1 μM FeCl_2_ or 1 μM MnCl_2_ and SOD activity was assessed in cell lysates (24.8 μg of total protein), with and without peroxide treatment. Peroxide treatment was used to inactivate Fe-containing SODs. Purified SodM (0.3 μg), loaded with either Mn or Fe, were included as controls. The experiment was repeated 3 times, and representative gels are shown.

The cambialistic properties of SodM raise the possibility that in Mn-deficient conditions, including those induced by the presence of CP, Fe-loaded SodM predominates in the cell. We took advantage of the fact that Fe-dependent SODs can be selectively inactivated by hydrogen peroxide to evaluate if both the Mn- and Fe-loaded forms of SodM are present in *S*. *aureus* [31]. For these experiments, SodM was expressed from a plasmid in Δ*sodA*Δ*sodM* and SOD activity was assessed following growth in Fe- and Mn-replete media. Control experiments using purified protein confirmed that the Fe-loaded form of SodM, but not the Mn-loaded forms of SodA or SodM, is sensitive to peroxide poisoning (Fig 2A). Consistent with prior studies [43], following growth in Mn-replete media SodM activity was not affected by peroxide indicating that the protein is loaded with Mn. However, when grown in Fe-replete media SodM activity was sensitive to hydrogen peroxide, indicating that it was Fe-loaded (Fig 4). These results indicate that in *S*. *aureus* SodM can be active with either Mn or Fe. CP has been observed to bind Fe^2+^, although it is unclear if this binding contributes to antimicrobial activity [13, 47]. As such, it raises the possibility that when exposed to CP *S*. *aureus* may be incapable of populating SodM with Fe. To evaluate the metallation state of SodM, lysates from cells cultured in the presence of CP were treated with H_2_O_2_ and assayed for SOD activity. In the absence of CP there was no reduction in activity associated with the heterodimer following peroxide treatment indicating that SodM is loaded with Mn. However, in the presence of CP, peroxide treatment eliminated almost all SodM activity, indicating that it is loaded with Fe (Fig 2A). Oxidative stress did not change the metal that was associated with SodM in both the presence and absence of CP (S1B Fig). Cumulatively, these observations suggest that the cambialistic nature of SodM enables *S*. *aureus* to resist host-imposed Mn starvation by facilitating the retention of SOD activity.

### SodM enhances the ability of *S*. *aureus* to resist Mn starvation during infection

**Fig 5:**
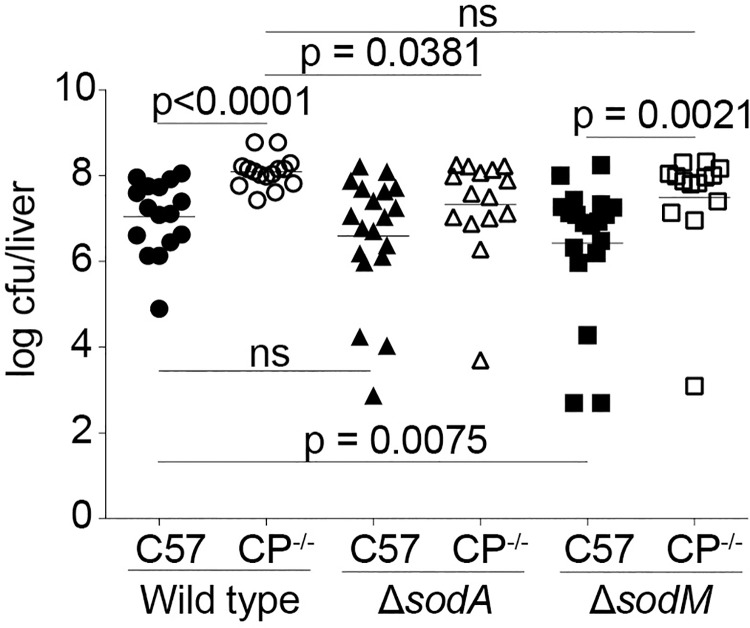
SodM contributes to resisting Mn starvation during infection. Wild type C57BL/6 (C57) and C57BL/6 S100A9^-/-^ (CP^-/-^) mice were infected with either wild type, Δ*sodA* or Δ*sodM S*. *aureus*. Mice were sacrificed 96 h after infection, and bacterial loads in the livers were enumerated. p <0.05 as determined by Mann-Whitney test.

In order to evaluate if SodA or SodM differentially contribute to pathogenesis based on Mn abundance during infection, we took advantage of the difference in Mn availability in wild type and CP-deficient mice [9]. Initially, the respective contributions of SodA and SodM to systemic disease in wild type C57BL/6 mice, in which the staphylococcal abscess is devoid of Mn, was assessed. In wild type mice, infection with Δ*sodA* resulted in a modest, but not significant, reduction in bacterial burden when compared to wild type *S*. *aureus*. In contrast, in wild type mice infected with Δ*sodM* there was a significant reduction in bacterial burden relative to wild type *S*. *aureus* (Fig 5), indicating that in the absence of Mn, SodM is critical for staphylococcal infection. Next, we infected CP-deficient mice, which fail to sequester Mn from staphylococcal liver abscesses [9]. Consistent with prior results, higher bacterial burdens were recovered from CP-deficient mice (C57BL/6 S100A9-/-) than wild type C57BL/6 mice infected with wild type *S*. *aureus* [9] (Fig 5). CP-deficient mice infected with Δ*sodA* had significantly reduced bacterial burdens when compared to wild type *S*. *aureus*. This result contrasts with CP-deficient mice infected with Δ*sodM*, which had bacterial burdens comparable to that of those infected with wild type *S*. *aureus*. In total, these observations indicate that SodA, but not SodM, contributes to staphylococcal disease when Mn is abundant. They also support the hypothesis that SodM contributes to the ability of *S*. *aureus* to resist host-imposed Mn starvation during infection.

## Discussion

During infection the innate immune system combats invading microbes by restricting the availability of the essential nutrient Mn [9, 12, 14]. At the same time, *S*. *aureus* and other pathogens must also overcome other host defenses including the oxidative burst of immune cells [23]. Accomplishing this latter task is made more challenging, as host-imposed Mn starvation inactivates bacterial Mn-dependent SODs [14]. The current investigations revealed that the possession of a cambialistic SOD enables *S*. *aureus* to counter these dual host threats both in culture and during infection. This strategy represents an entirely new mechanism for resisting host-imposed Mn starvation and establishes that cambialistic SODs contribute to bacterial pathogenesis.

The Fe/Mn superfamily of SODs is widely distributed in bacteria, archaea, and eukaryotes. Members of this family are generally thought to be reliant on either Mn or Fe for catalytic activity [28, 30, 48]. However, since the 1980s, predominantly *in vitro* analyses have suggested that a subset of these enzymes, termed cambialistic SODs, are capable of using both Fe and Mn, [32–34, 38–40]. Cambialistic SODs have been reported in both Gram-positive and Gram-negative bacteria, including the human pathogens *Porphyromonas gingivalis*, *Streptococcus pneumoniae*, and *Streptococcus mutans* and suggested to be present in other microbes including *Bacteroides fragilis*, and *Bacteroides thetaiotaomicron* [32–40, 49–52]. However, the lack of detailed *in vivo* studies and the fact that many cambialistic SODs have greater activity when loaded with one or the other cofactor *in vitro* has resulted in skepticism regarding the importance of cambialism [32, 36, 38–40]. As such, a false dichotomy exists that members of the Fe/Mn SOD family must use either Mn or Fe but not both. This dichotomy has led to confusion over the biologically relevant metal utilized by several bacterial SODs, especially given the difficulty in predicting the cofactor utilized by this family of enzymes using bioinformatics [28]. The observation that SodM has equal activity with either Mn or Fe *in vitro*, can be activated with both metals *in vivo*, and promotes resistance to nutritional immunity during infection establishes a clear and important role for cambialistic SODs in facilitating resistance to host defenses. Given the ubiquity of CP and host-imposed metal starvation during infection, it seems likely that expression of a cambialistic SOD would provide a benefit to other pathogens as well. Cambialistic SODs are also found in a diverse collection of environmental microbes [32, 33, 35, 39], suggesting cambialism may represent a generalized strategy used by organisms to maintain a defense against superoxide in niches where Fe and Mn availability can fluctuate.

While metal-dependent mononuclear enzymes have historically been thought to utilize a specific cofactor, it has become apparent that there can be significant plasticity in the metal cofactor they can utilize, particularly in the case of Mn- and Fe-utilizing enzymes [53]. In response to peroxide stress *E*. *coli* and many other pathogens sequester intracellular Fe and increase the expression of Mn importers, which in turn leads to accumulation of this metal [53–55]. In *E*. *coli*, this action results in Fe-utilizing enzymes, such as ribulose-5-phosphate 3-epimerase, becoming populated with Mn [53, 56, 57]. This change in cofactors enables *E*. *coli* to both maintain enzymatic activity and prevent Fenton chemistry-induced damage, which can arise from the interaction of Fe^2+^ with oxidants [53, 56, 57]. Notably, in many Fe-centric organisms, including *E*. *coli*, *Salmonella typhimurium*, and *Yersinia pestis*, Fe starvation increases the expression of Mn uptake systems and the accumulation of Mn [54, 58–60]. In addition to enabling bacteria to activate Mn-dependent isozymes [61], the increased Mn levels may also allow them to replace Fe with Mn in non-redox enzymes. These observations in conjunction with our findings suggest that populating metalloenzymes with an alternative yet catalytically active metal may be a general strategy used by bacteria to survive when a specific metal is limiting. While specific examples for metals other than Fe and Mn are currently lacking, conceptually this cofactor plasticity may enable microbes to maintain critical metabolic processes when limited for other essential metals.

Amongst the staphylococci, *S*. *aureus* is the most pathogenic species and the only one that expresses two SODs [41–44], with SodM presumably being gained through duplication and subsequent divergence. However, the advantage that *S*. *aureus* gains by expressing two Mn-dependent SODs, which are 75% identical at the level of their primary sequence, had not been apparent. Our current studies found that SodM is induced by CP-imposed Mn-starvation. The observation that SodM is cambialistic and enables *S*. *aureus* to maintain SOD activity when Mn starved by the host provides a rationale for its acquisition. It also suggests a model in which the Mn-dependent SodA is important during the initial colonization of a tissue, while SodM becomes important later during infection following the imposition of Mn starvation by the host immune response. Notably, *S*. *aureus* is not the only pathogen to express multiple superoxide dismutases that initially appear to be functionally redundant in culture, but upon subsequent analysis possesses properties that enhance fitness in the context of pathogenesis [62, 63]. For example, a second Cu/Zn SOD expressed by some *Salmonella* is protease-resistant and binds to peptidoglycan, which enables it to retain activity and promote survival within the phagolysosome [63]. Cumulatively, these observations and our results emphasize the importance of evaluating the contribution of apparently redundant SODs to resisting oxidative stress in the context of the other stressors that an organism encounters within its ecological niche.

The antimicrobial activity of CP is generally thought to be mediated by the sequestration of Mn^2+^ and Zn^2+^ [9, 20, 21]. However, CP was recently shown to bind Fe^2+^, resulting in the suggestion that Fe restriction is a primary driver of its antimicrobial activity [47]. Notably, CP does not bind Fe^3+^, the ionic state that exists in oxidizing environments such as sites of infection [9, 20, 47]. Additionally, several experimental lines of evidence suggest that Mn limitation contributes to the antimicrobial activity of CP both in culture and during infection. In both *Acinetobacter baumannii* and *S*. *aureus*, CP reduces intracellular Mn but not Fe levels [17, 64]. The current observation that *S*. *aureus* replaces Mn in SodM with Fe even in the presence of concentrations of CP approaching 1 mg/ml further supports the idea that in culture CP is not imposing Fe limitation on *S*. *aureus*. Furthermore, in wild type mice loss of MntABC and MntH, the two Mn importers expressed by *S*. *aureus*, results in a substantial reduction in virulence; however, this defect is completely reversed in CP-deficient mice [13]. The observation that SodM is critical for infection in wild type but not CP-deficient mice further supports the idea that Mn but not Fe sequestration by CP contributes to controlling infection. Perhaps not surprisingly, given the myriad of high affinity staphylococcal Fe acquisition systems [65], it also suggests that during infection *S*. *aureus* more successfully competes with the host for Fe than Mn. Cumulatively, these findings strongly support, at least in the case of *S*. *aureus* and *A*. *baumannii*, that Mn and not Fe sequestration significantly contributes to the antimicrobial activity of CP.

Antibiotic resistance is a serious and growing threat to human health, with multiple agencies calling for the development of new approaches to treat bacterial infections [1, 2]. Understanding how pathogens overcome innate immune defenses has the potential to reveal new opportunities for therapeutic intervention. Our studies reveal a new mechanism by which bacteria can overcome a two-pronged attack by the host. They also clearly demonstrate a role for cambialism in resisting nutritional immunity and bacterial pathogenesis. Moreover, these results provide newfound importance for a neglected family of proteins that is widely distributed throughout the tree of life.

## Methods

### Ethics Statement

All animal work was approved by the Vanderbilt University Institutional Animal Care and Use Committee (protocol #M1600123) and was performed in accordance with United States Public Health Service Policy on Humane Care and Use of Laboratory Animals and the US Animal Welfare Act.

### Bacterial Strains

*Staphylococcus aureus* strain Newman was used unless otherwise indicated. All strains and plasmids used in this study are listed in Tables 1 and 2. *S*. *aureus* was routinely grown in tryptic soy broth (TSB) and on tryptic soy agar plates (TSA), while *E*. *coli* was routinely cultivated in Luria Broth (LB) and on Luria agar plates. Both species were grown at 37°C. As needed for plasmid maintenance or gene inductions, 10 μg/ml of chloramphenicol, 50 μg/ml of kanamycin, 100 μg/ml of ampicillin, or 10 ng/ml of anhydrotetracycline was included in the media used. All strains were stored at -80°C in media containing 30% glycerol.

**Table 1:** Strains used in this study.

Strain	Description	Reference
Wild type	*S*. *aureus* Newman	
*ΔsodA*	*S*. *aureus* Newman *sodA*::*tet*	This Study
*ΔsodM*	*S*. *aureus* Newman *sodM*::*erm*	This Study
*ΔsodA*Δ*sodM*	*S*. *aureus* Newman *sodA*::*tet sodM*::*erm*	[14]
WT pRMC2	*S*. *aureus* Newman carrying pRMC2	This study
*ΔsodA*Δ*sodM* pRMC2	Δ*sodA*Δ*sodM* carrying pRMC2	This study
*ΔsodA*Δ*sodM* pRMC2-*sodA*	*ΔsodA*Δ*sodM* carrying pRMC2-*sodA*	This study
*ΔsodA*Δ*sodM* pRMC2-*sodM*	*ΔsodA*Δ*sodM* carrying pRMC2-*sodM*	This study
WT pEmpty	*S*. *aureus* Newman carrying pAH5-empty	This study
WT pSodA	*S*. *aureus* Newman carrying pAH5-p*sodA*	This study
WT pSodM	*S*. *aureus* Newman carrying pAH5-p*sodM*	This study

**Table 2:** Plasmids used in this study.

Plasmid	Description	Reference
pRMC2	Anhydrotetracycline-inducible plasmid	[66]
pRMC2-*sodA*	*sodA* cloned into pRMC2	This study
pRMC2-*sodM*	*sodM* cloned into pRMC2	This study
pAH5	YFP reporter plasmid	[67]
pAH5-p*sodA*	pAH5 with the *sodA* promoter driving YFP expression	This study
pAH5-p*sodM*	pAH5 with the *sodM* promoter driving YFP expression	This study
pEmpty	pAH5 without a promoter driving YFP expression	This study
pET29a-*sodA*	SodA expression vector	This study
pET29a-*sodM*	SodM expression vector	This study

### Construction of staphylococcal mutants and plasmids

The *ΔsodA* and *ΔsodM* mutants were created via Phi85 transduction of the *sodA*::tet and *sodM*::erm alleles from RN6390 [43]. The staphylococcal SodA and SodM expression constructs were created by amplifying *sodA* and *sodM* using the primers indicated in Table 3 and then cloning these fragments into the anhydrotetracycline-inducible plasmid pRMC2 [66]. To generate the YFP reporter plasmids the promoters for *sodA* and *sodM* were amplified with the primers listed in Table 3 and then cloned using standard techniques into pAH5 [67]. To generate the promoterless YFP construct pAH5 was digested with *Pst*I and *Kpn*I to remove the existing promoter, blunted and then self-ligated.

**Table 3:** Primers used in this study. Underlined bases represent restriction sites used for subcloning, and bases highlighted in bold represent mutagenized bases.

Name	Sequence
sodA 5’ cloning:	cgatagatctccagtcactgcttgttattatttacttacagac
sodA 3’ cloning:	cagtgaattccggtctcatttaagagaccgaacaag
sodM 5’ cloning:	cgatagatctcataaaaggaggaatatacttatggcatttaaattac
sodM 3’ cloning:	cagtgaattcgaacaccttgtagatgctccacc
sodA 5’ reporter:	cgatggatccgaagtttatggtgtatgtgagtcttgcc
sodA 3’ reporter:	cagtggtaccaaataatcatcctcctaaaatgtctgtaag
sodM 5’ reporter:	cgatggatccgtaccggtaatgctatactttagaaaattaag
sodM 3’ reporter:	cagtggtaccaagtatattcctccttttatgaatatacttttataataattaatttcg
sodA_for:	Gggcatatggcttttgaattacc
sodA_rev:	ggggatccttattttgttgcattatataattcg
sodM_for:	gggcatatggcatttaaattacc
sodM_rev:	gggatcctattattttgctgcttgg
sodMqc_for:	taaattaccaaatttaccata**c**gc**g**tatgatgcattggaaccatat
sodMqc_rev:	atatggttccaatgcatcata**c**gc**g**tatggtaaatttggtaattta
sodMqc1_for:	ggaacattgttaactggaaaaa**a**gttgatgaattataccaagcagc
sodMqc1_rev:	gctgcttggtataattcatcaac**t**tttttccagttaacaatgttcc

### CP growth assays

Calprotectin growth assays were performed largely as previously described [13, 20], with the exception that overnight cultures were performed in Chelex-treated RPMI + 1% casamino acids (NRPMI) supplemented with 1 mM MgCl_2_, 100 μM CaCl_2_, and 1 μM FeCl_2_. These cultures were diluted 1:100 into 100 μl of culture medium in a 96-well round-bottom plate and incubated at 37°C and with shaking at 180 rpm. The culture medium consisted of 38% TSB and 62% CP buffer (3 mM CaCl_2_, 20mM Tris base, and 100 mM NaCl, 10 mM β-mercaptoethanol, pH 7.5) supplemented with 1 μM MnCl_2_ and 1 μM ZnSO_4_. Where indicated 0.1 mM PQ was added to the media. The same growth conditions were utilized for the expression studies. For both growth and expression assays, optical density (OD_600_) and fluorescence was assessed after 8 hrs of growth.

### SOD activity

Total and individual superoxide dismutase (SOD) activity were assayed using a water-soluble tetrazolium salt assay and a gel-based nitro blue tetrazolium assay, respectively, as previously described [14, 68]. For both assays, the bacteria were grown as for the CP assays and harvested in exponential phase (OD_600_ of ~ 0.3–0.35). The cells were collected and then resuspended in 0.5 mM KPO_4_ buffer at pH 7.8 with 0.1 mM EDTA [46]. The bacteria were then lysed via mechanical disruption and centrifuged to remove insoluble material. The protein concentration in the cell lysate was determined via BCA assay (Pierce). Total SOD activity was assessed using the SOD Assay Kit (Sigma-Aldrich), per the manufacturer’s instructions. To evaluate the individual activity of each SOD, the cell lysates were normalized to total protein concentration and resolved on 10% native polyacrylamide gel. The gels were incubated in buffer containing 0.05 M potassium phosphate pH 7.8, 1 mM EDTA, 0.25 mM nitro blue tetrazolium chloride, and 0.05 mM riboflavin and then exposed to light, as previously described [68]. To evaluate if SOD activity was due to iron-loading, prior to assessing activity the gels were incubated with 20 mM H_2_O_2_ or water for 20 minutes. Gels were imaged using a BioRad imager Universal Hood II and the fractional distribution of SOD activity was determined using the BioRad Quantity One software.

### Cloning of the *sodA* and *sodM* genes for heterologous expression

The *sodA* and *sodM* genes were amplified by PCR from *S*. *aureus* genomic DNA using *Pfu* polymerase (NEB) and the primer pairs sodA_for and sodA_rev and sodM_for and sodM_rev, respectively, which incorporated 5’ *Nde*I and 3’ *Bam*HI restriction sites. PCR products were A-tailed with *Taq* polymerase (NEB) and cloned into the pGEM-T vector (Promega) to yield pGEM-T-sodA and pGEM-T-sodM, respectively. An internal *Nde*I site in the *sodM* sequence was silently mutated by site-directed mutagenesis using the primer pair sodMqc1_for and sodMqc1_rev to yield pGEM-T-sodMqc. The genes were sub-cloned through *Nde*I/*Bam*HI (NEB) digestion of the pGEM-T constructs, purification of the gene inserts by agarose gel electrophoresis, and subsequent ligation into *Nde*I/*Bam*HI-digested pET29a vector (Novagen) to yield pET29a-*sodA* and pET29a-*sodM* constructs. Both pET29a constructs were sequenced (GATC Biotech, Germany). A sequence error detected in pET29a-*sodM* (deletion of base A570) was subsequently corrected through site-directed mutagenesis using primers sodMqc2_for and sodMqc2_rev, and the final construct confirmed through sequencing.

### Expression, purification and quantification of recombinant SodA and SodM

The pET29a-sodA and pET29a-sodM constructs were transformed into *Escherichia coli* BL21 (λDE3) cells and selected on LB agar plates containing 50 μg/ml kanamycin. For each cell type, cells were inoculated into M9 medium containing 10 μM FeSO_4_ and 50 μg/ml kanamycin and cultured overnight at 37°C with 180 rpm orbital shaking. This overnight culture was used to inoculate 1 L M9 medium containing 10 μM FeSO_4_ and 50 μg/ml kanamycin, cultured at 37°C with 180 rpm orbital shaking. At OD_600_ ~0.5, protein expression was induced by addition of 1 mM isopropyl β–D-1-thiogalactopyranoside (IPTG) plus a further 20 μM FeSO_4_ and incubation for 4 h under the same conditions. Cells were harvested by centrifugation (20 min, 4,000 g, 4°C), washed in 20 mM Tris(hydroxymethyl)aminomethane (Tris), pH 7.5, 10 mM ethylenediaminetetraacetic acid (EDTA), followed by a further wash in 20 mM Tris, pH 7.5, 150 mM NaCl and stored at -20°C.

Cells were resuspended in 20 mM Tris, pH 7.5 and lysed by sonication (6 x 10 s, with 1 min intervals, on ice) and the lysate clarified by centrifugation (20 min, 19,000 g, 4°C). The soluble lysate was loaded onto a 5 ml HiTrap Q HP anion exchange chromatography (AEC) column (GE Healthcare), the column was washed with 5 column volumes (CV) of buffer (20 mM Tris, pH 7.5), followed by elution with a 9 CV linear NaCl gradient (0–1 M NaCl) in the same buffer, collecting 2 ml fractions, using an Äkta fast performance liquid chromatography (FPLC) system (GE Healthcare). Fractions were analyzed for protein by sodium dodecyl sulfate polyacrylamide gel electrophoresis (SDS-PAGE). SodA eluted from AEC at ~230 mM NaCl, whereas SodM eluted at ~248 mM NaCl. Aliquots (1 ml) of the peak AEC fractions containing the recombinant protein were further purified using the Äkta FPLC by size exclusion chromatography (SEC) on a Superdex 200 16/60 column (GE Healthcare), resolved in 20 mM Tris, 150 mM NaCl, 5 mM EDTA, pH 7.5 at 1 ml/min, collecting 2 ml fractions.

Concentrations of purified recombinant SodA and SodM were determined from A_280nm_ measurements, using the empirically determined extinction coefficients (ε_280nm_) of 62,681 M^-1^ cm^-1^ for SodA and 64,949 M^-1^ cm^-1^ for SodM, each derived from quantitative amino acid analysis (Alta Bioscience, UK). The metal content of the purified proteins was assessed by ICP-MS, as described below.

### Unfolding and refolding of recombinant SOD proteins

To reconstitute recombinant SodA and SodM, which contained primarily Fe when purified from *E*. *coli*, with exclusively Mn they were unfolded and refolded in excess Mn, as previously described [69], with modifications. Unfolding was performed in 2.5 M guanidine hydrochloride, in the presence of 5 mM EDTA and 20 mM 8-hydroxyquinoline to remove bound metal ions, followed by refolding through several rounds of dialysis against 20 mM Tris, 100 mM NaCl, 10 mM MnCl_2_, pH 7.5, to yield protein containing exclusively manganese. To analyze the bound metal, aliquots of each purified protein (~2 mg in 0.5 ml) were resolved on a Superdex 200 Increase 10/30 column (GE Healthcare) in 20 mM Tris, 150 mM NaCl, 5 mM EDTA, pH 7.5, resolved at 0.75 ml/min and collecting 0.5 ml fractions using the Äkta FPLC. Eluant fractions were analyzed for protein by A_280nm_ and by SDS-PAGE, for elemental composition by ICP-MS, and for enzyme activity by in-gel activity assay.

### Elemental analysis by inductively coupled plasma mass spectrometry

For elemental analysis via ICP-MS, protein-containing samples were diluted 50-fold into a solution of 2.5% HNO_3_ (Suprapur, Merck) containing 20 μg/l Co and Pt as internal standards. Matrix-matched elemental standards (containing analyte metal concentrations of 0–500 μg/L) were prepared by serial dilution from individual metal standard stocks (VWR) with identical solution compositions, including the internal standard. All standards and samples were analyzed by ICP-MS using a Thermo x-series instrument operating in collision cell mode (using 3.0 ml/min flow of 8% H_2_ in He as the collision gas). Isotopes ^55^Mn, ^56^Fe, ^59^Co, ^66^Zn, and ^195^Pt were monitored using the peak-jump method (100 sweeps, 20–30 ms dwell time on 3–5 channels per isotope, separated by 0.02 atomic mass units) in triplicate, and metal concentrations determined from the standard curve.

### Animal infections

Nine-week-old female black C57BL/6 and CP -/- (C57BL/6 S100A9-/-) mice were infected using a retro-orbital infection model [9, 13, 14]. Livers were harvested and homogenized 96 hours post-infection. Serial dilutions were then plated and counted for colony forming units.

## Supporting Information

S1 Fig SodM is the predominant source of SOD activity in Mn-deplete environments regardless of oxidative stress. Wild type *S*. *aureus* and Δ*sodA*Δ*sodM* were grown in the presence of CP, in the (A-C) presence and (A & B) absence of 0.1 mM PQ and (A) total SOD activity and (C) the individual contributions of SodA and SodM to SOD activity were determined (n = 3). (B) In-gel analysis of the individual activities of SodA and SodM following growth in the presence of CP and PQ. Hydrogen peroxide treatment was used to inactivate Fe-containing SODs. The experiment was repeated 3 times and representative gels are shown. * = p <0.05 relative to no CP via two-way ANOVA with Tukey’s post-test.
(TIF)

S2 Fig Contribution of SodA and SodM to resisting oxidative stress and metal limitation. (A & B) The Δ*sodA***Δ***sodM* mutant expressing either SodA or SodM from a plasmid was grown in various concentrations of CP in the (A) absence and (B) presence of 0.1 mM PQ. Growth (OD_600_) was measured after 8 h. * = p <0.05 relative to WT containing empty vector via two-way ANOVA with Dunnett’s post-test. Error bars indicate SEM (n = 3 or more). (C-D) Wild type *S*. *aureus*, Δ*sodA*, Δ*sodM*, and Δ*sodA*Δ*sodM* were grown in the presence of various concentrations of (C) the ΔMn/Zn site CP mutant or (D) the ΔZn site mutant in the absence of PQ. * = p <0.05 relative to wild type at the same concentration of CP via two-way ANOVA with Dunnett’s post-test. Error bars indicate SEM (n = 3 or more).
(TIF)

S3 Fig Elemental analysis of purified and remetallated SodA and SodM. Aliquots **(**~2 mg in 0.5 ml) of purified recombinant (A) SodA and (B) SodM were resolved by analytical size exclusion chromatography and eluant fractions (0.5 ml) were analyzed for protein by A_280nm_ (black), and for manganese (blue) and iron (red) content by ICP-MS. Both proteins contained exclusively iron when purified from the heterologous host. Each protein was then unfolded, stripped of iron, and refolded in the presence of manganese. The resulting proteins were analyzed identically, and the refolded (C) SodA and (D) SodM were found to contain exclusively manganese. (E) Each of the four resulting samples (Fe-SodA, Fe-SodM, Mn-SodA and Mn-SodM) were subjected to protein analysis by SDS-PAGE, and to both in-gel and spectrophotometric SOD activity analysis.
(TIF)
