# Supplementary figures and images for: A Superoxide Dismutase Capable of Functioning with Iron or Manganese Promotes the Resistance of Staphylococcus aureus to Calprotectin and Nutritional Immunity
Source: PLoS Pathog. 2017 Jan 19;13(1):e1006125. doi: 10.1371/journal.ppat.1006125 (PMC5245786; doi:10.1371/journal.ppat.1006125)

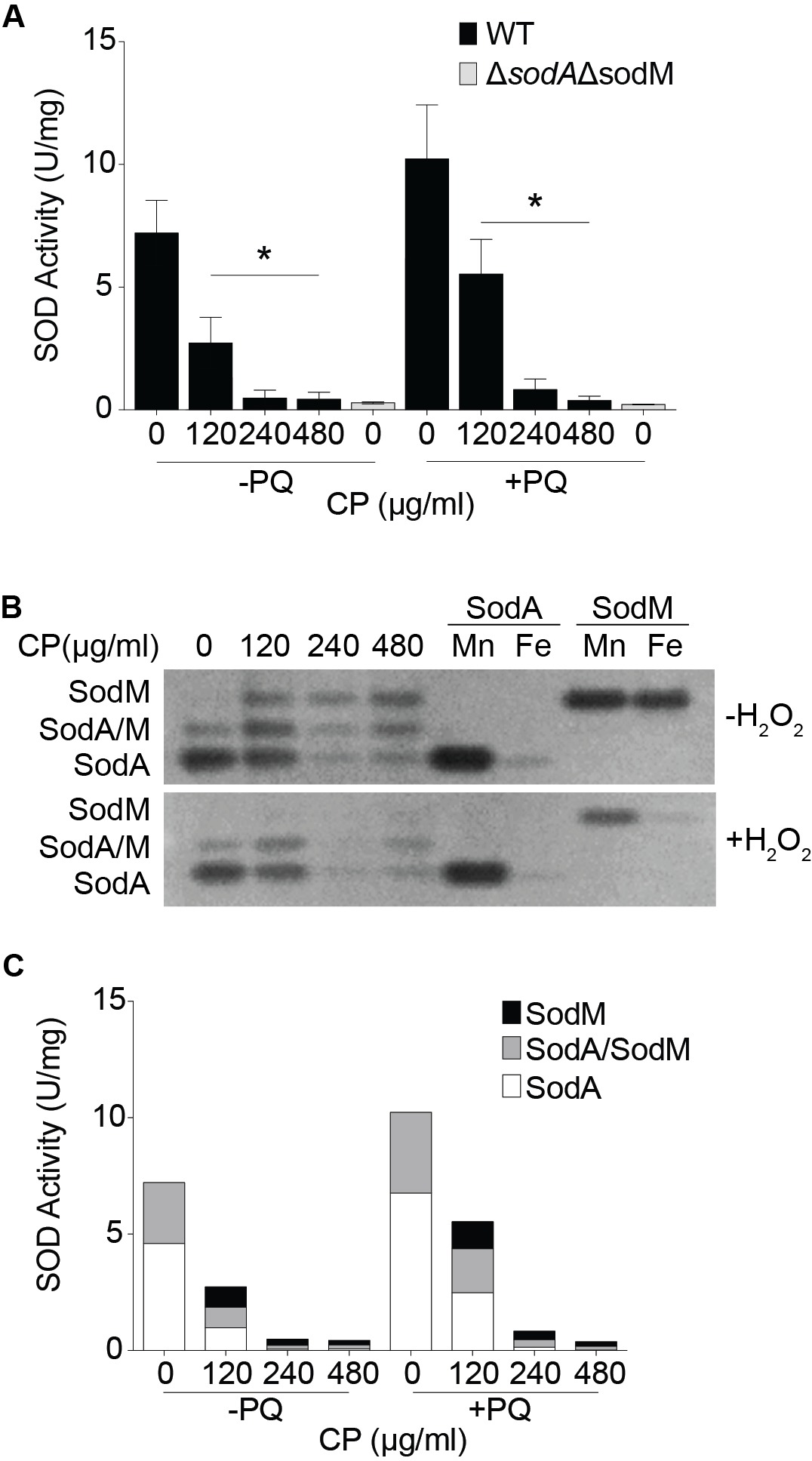

Supplement: S1 Fig — Wild type S. aureus and ΔsodAΔsodM were grown in the presence of CP, in the (A-C) presence and (A & B) absence of 0.1 mM PQ and (A) total SOD activity and (C) the individual contributions of SodA and SodM to SOD activity were determined (n = 3). (B) In-gel analysis of the individual activities of SodA and SodM following growth in the presence of CP and PQ. Hydrogen peroxide treatment was used to inactivate Fe-containing SODs. The experiment was repeated 3 times and representative gels are shown. * = p <0.05 relative to no CP via two-way ANOVA with Tukey’s post-test. (TIF) [file ppat.1006125.s001.tif]

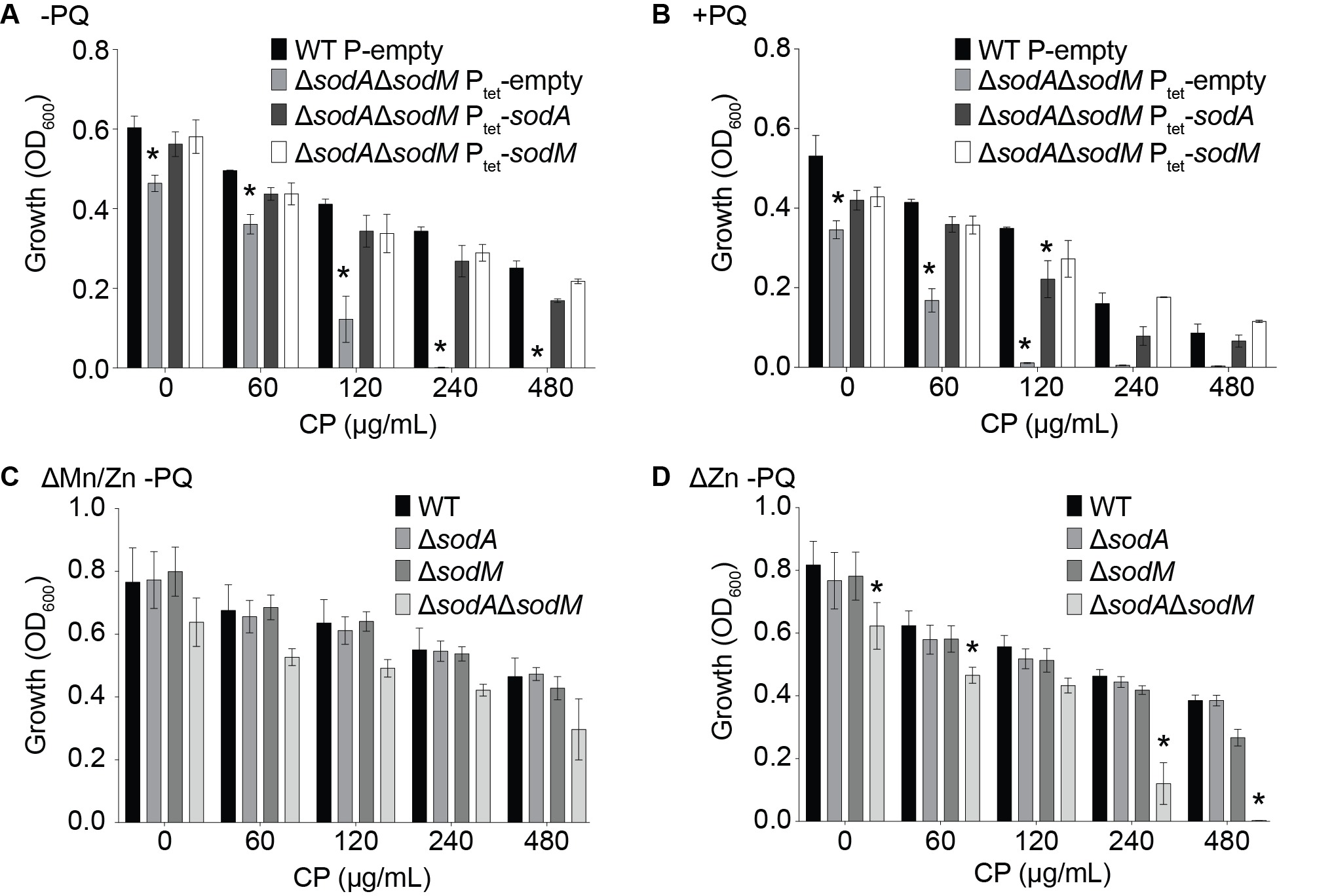

Supplement: S2 Fig — (A & B) The ΔsodAΔsodM mutant expressing either SodA or SodM from a plasmid was grown in various concentrations of CP in the (A) absence and (B) presence of 0.1 mM PQ. Growth (OD600) was measured after 8 h. * = p <0.05 relative to WT containing empty vector via two-way ANOVA with Dunnett’s post-test. Error bars indicate SEM (n = 3 or more). (C-D) Wild type S. aureus, ΔsodA, ΔsodM, and ΔsodAΔsodM were grown in the presence of various concentrations of (C) the ΔMn/Zn site CP mutant or (D) the ΔZn site mutant in the absence of PQ. * = p <0.05 relative to wild type at the same concentration of CP via two-way ANOVA with Dunnett’s post-test. Error bars indicate SEM (n = 3 or more). (TIF) [file ppat.1006125.s002.tif]

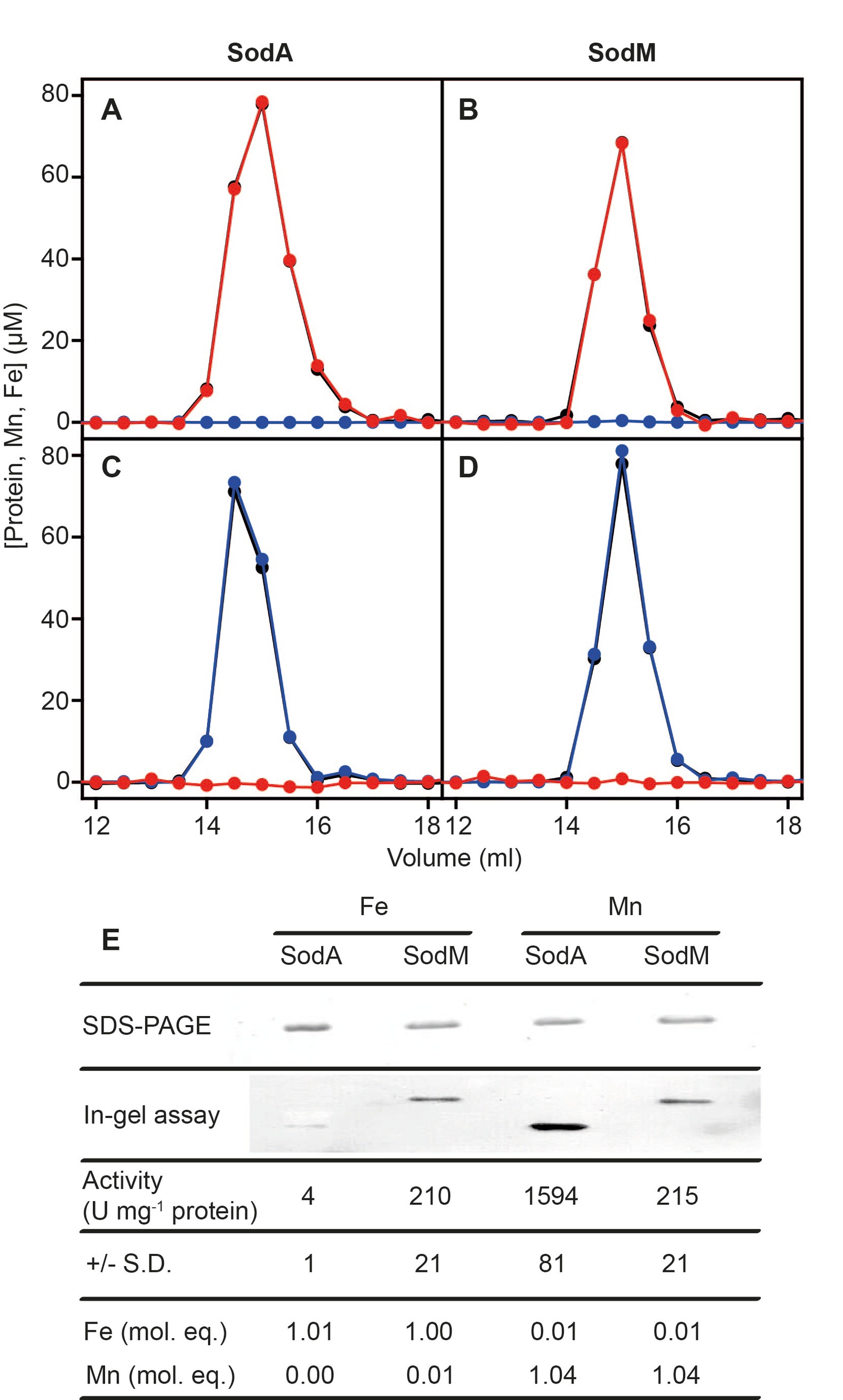

Supplement: S3 Fig — Aliquots (~2 mg in 0.5 ml) of purified recombinant (A) SodA and (B) SodM were resolved by analytical size exclusion chromatography and eluant fractions (0.5 ml) were analyzed for protein by A280nm (black), and for manganese (blue) and iron (red) content by ICP-MS. Both proteins contained exclusively iron when purified from the heterologous host. Each protein was then unfolded, stripped of iron, and refolded in the presence of manganese. The resulting proteins were analyzed identically, and the refolded (C) SodA and (D) SodM were found to contain exclusively manganese. (E) Each of the four resulting samples (Fe-SodA, Fe-SodM, Mn-SodA and Mn-SodM) were subjected to protein analysis by SDS-PAGE, and to both in-gel and spectrophotometric SOD activity analysis. (TIF) [file ppat.1006125.s003.tif]
